# Students’ experience of the Health Care Team Challenge™: long-term case competition can improve students’ competence in interprofessional collaboration

**Published:** 2013-09-30

**Authors:** Daniel Ting, Amanda Sutherland, Catherine Donnelly

**Affiliations:** 1 School of Medicine, Queen’s University, Kingston, Ontario, Canada; 2 School of Rehabilitation Therapy, Queen’s University, Kingston, Ontario, Canada

## Introduction

In Ontario, recent policy documents have considered interprofessional (IP) care as one of the cornerstones of the health care system.^1^,^2^ Nevertheless, many healthcare workers lack awareness about how to work effectively in IP teams, and hence fail to fully capitalize on its benefits.^3^ To improve competency in collaboration, Canadian health sciences programs have widely introduced IP education as part of their curricula. One IP education strategy is the Health Care Team Challenge (HCTC), a multidisciplinary case competition.^4^,^5^ Recent reports have identified key characteristics from an organizer’s point of view,^6^,^7^ and have shown statistically significant improvement in students’ attitudes towards IP collaboration.^7^ Nevertheless, there has been little formal feedback from the participants’ perspective. Unanswered questions include the process of team development and learning for students, and whether the HCTC influences students’ attitudes towards IP collaboration.^6^

As a group of professional trainees and a faculty mentor (with backgrounds in medicine, nursing, physical therapy and occupational therapy), we report on our experience working as an IP team during the 2011–2012 regional and national Canadian HCTC competitions.

## The Health Care Team Challenge

The University of British Columbia first introduced the HCTC as a local event in the 1980s as a solution to the lack of IP interaction and collaboration among health sciences students. The HCTC has since been expanded to various institutions on the international stage.^6^ The Canadian national competition involves two stages: a qualification event at the institutional level, and nationals, which in 2012 were held in Kitchener, Ontario. For qualifying teams, the IP experience spans an academic year. At both levels of the challenge, teams are given three weeks to prepare an IP care plan for a complex case study. During competition day, teams present orally to a panel of judges and other live audience members comprised of clinicians, academics, community members, and students. In addition to presenting prepared questions, teams are tested with impromptu problems. Responses are evaluated based on the quality of the management plan, creativity, and the degree of IP collaboration.^5^

### Participant Experiences

The Canadian Interprofessional Health Collaborative has defined IP competency as a framework that includes communication, team functioning, leadership and role clarification.^8^ We seek to further explore three features of the competition that we identified during group reflection to be the most important for meeting these competency goals, which in turn improved attitudes towards IP practice.

First, regular face-to-face meetings over several months established a common language devoid of profession-specific jargon. Such lessons included avoiding abbreviations whenever possible, and pausing at various points to clarify ideas and understand differing perspectives. Our progress on cases was kept in a central Google document, and the note taker during sessions was rotated so that the language would remain neutral and not profession-specific.

Second, a dedicated faculty mentor anchored team discussions to real life scenarios by providing insight and experience. For instance, when our team questioned how professionals could communicate face-to-face in real life, our mentor introduced us to the concept of interprofessional board rounds as regularly scheduled activities at Kingston General Hospital, which are principal events to communicate issues to different professionals. These insights helped motivate the team to produce pragmatic solutions. The mentor’s presence also deepened her insight into student IP collaboration, such as the need to quickly establish a nonhierarchical team environment.

Third, the challenge itself encouraged team unification and led to collaborative leadership when developing treatment plans for the patient. For instance, the occupational therapy and physical therapy students consulted with the medical and nursing students to determine whether the patient would be medically stable enough to participate in rehabilitation interventions. These discussions spurred us to learn more about different interprofessional roles, which helped team members recognize strengths of different professions and empowered members to take ownership of their areas of expertise. This development helped foster mature attitudes towards IP collaboration.

## Recommendation

We propose that in future iterations of the HCTC, case scenarios should be developed specifically to reflect the continuum of care, from acute care to long-term care to community settings. Our regional competition involved stroke management and rehabilitation, which was an excellent example that incorporated these various settings. Furthermore, the judging panel should have themselves collaborated together in real life and represent disciplines relevant to the case. At our national competition, the judging panel lacked any member from nursing or medicine, making it difficult to accurately judge the validity of the medical aspect of the care plan. Students would benefit further from having the judges create IP questions for each team at the conclusion of their presentations, instead of profession-specific queries.

One of the weaknesses of the HCTC model is that it is resource-intensive and therefore involves only a small fraction of the student population. At Queen’s University, there are shorter, mandatory events that involve all professional students, but which often are met with student apathy. We believe that making the events into a challenge and involving more faculty members would be effective ways to motivate team building and engagement.

Undoubtedly, the momentum of IP healthcare is building, and the need for effective educational strategies is pressing. We believe that the HCTC represents an opportunity for developing IP competence and should be expanded to include more students, more teams and more institutions.
